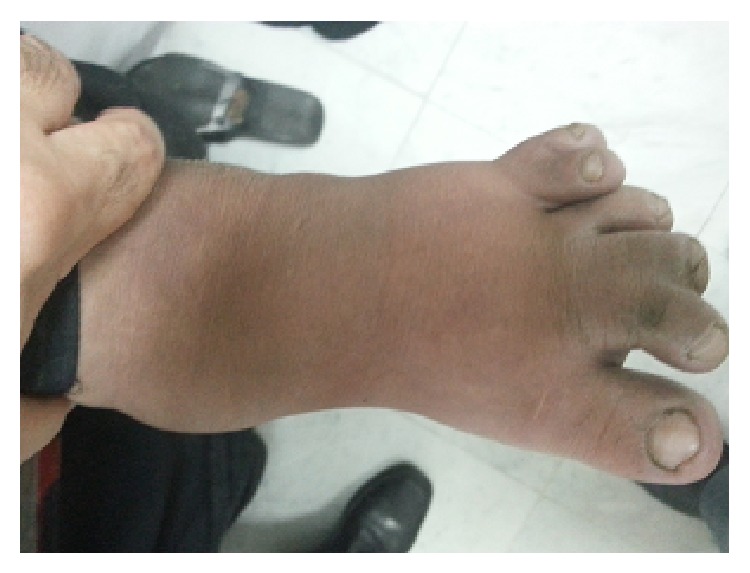# Nystagmus in Laurence-Moon-Biedl Syndrome

**DOI:** 10.1155/2015/439409

**Published:** 2015-04-23

**Authors:** A. Bruce Janati, Naif Saad ALGhasab, Fazal Haq, Ahmad Abdullah, Aboubaker Osman

**Affiliations:** ^1^Center for Neurology in Fairfax, VA, USA; ^2^King Faisal Specialist Hospital, UOH, P.O. Box 6252, Hail 81442, Saudi Arabia; ^3^King Khaled Hospital, Hail, Saudi Arabia

## Abstract

*Introduction*. Laurence-Moon-Biedl (LMB) syndrome is a rare autosomal-recessive ciliopathy with manifold symptomatology. The cardinal clinical features include retinitis pigmentosa, obesity, intellectual delay, polydactyly/syndactyly, and hypogenitalism. In this paper, the authors report on three siblings with Laurence-Moon-Biedl syndrome associated with a probable pseudocycloid form of congenital nystagmus. *Methods*. This was a case study conducted at King Khaled Hospital. *Results*. The authors assert that the nystagmus in Laurence-Moon-Biedl syndrome is essentially similar to idiopathic motor-defect nystagmus and the nystagmus seen in optic nerve hypoplasia, ocular albinism, and bilateral opacities of the ocular media. *Conclusion*. The data support the previous hypothesis that there is a common brain stem motor abnormality in sensory-defect and motor-defect nystagmus.

## 1. Introduction

Laurence-Moon-Biedl (LMB) syndrome is a rare autosomal-recessive ciliopathy with manifold symptomatology [1, 2]. The cardinal clinical features include retinitis pigmentosa, obesity, intellectual delay, polydactyly/syndactyly, and hypogenitalism. Although nystagmus has often been mentioned as a component of this syndrome, its characteristics have not been thoroughly investigated. In this paper, we report on three siblings with LMB who presented with a probable pseudocycloid type of congenital nystagmus.

## 2. Case Report

### 2.1. Case 1

**Figure 1:**
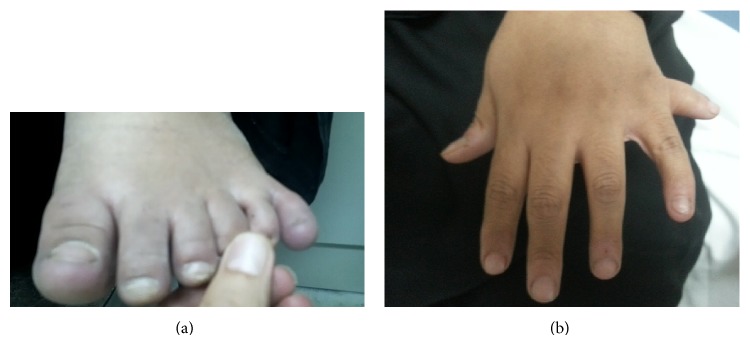


The patient was a 31-year-old female who presented with chronic progressive visual loss. There was no history of oscillopsia. She had exhibited intellectual delay with speech impediment and marked obesity. Her general physical examination showed only finger recognition in both eyes, truncal obesity, and retinitis pigmentosa. She had polydactyly (hexadactyly), syndactyly in both feet (Figure 1(a)), and polydactyly in the left hand (Figure 1(b)). She had a slow gait. Cranial nerves were intact. Motor system was normal. Deep tendon reflexes were depressed but symmetrical bilaterally. Cerebellar functions were normal. There was no Babinski sign. A conspicuous finding was a conjugate and horizontal nystagmus with a frequency of 4 cycles per second, which was accentuated by fixation attempts and attenuated by convergence. It was most prominent on attempted right and left lateral gaze. It consisted of smooth eye movements of increasing velocity away from the target followed by refoveating saccades. The “null point” was in the primary position. There were no abnormal head positions or head movements.

Systemic-metabolic work-up including endocrine tests was unremarkable. EEG and brain MRI were both normal.

### 2.2. Case 2

The patient was a 17-year-old female with intellectual delay, bilateral visual loss, and obesity. There was no history of oscillopsia. General physical examination showed predominantly truncal obesity, retinitis pigmentosa, and polydactyly (hexadactyly) and syndactyly in both feet (Figure 2). Visual acuity was limited to finger recognition in both eyes. She had an awkward gait. Neurological examination showed normal mental status and cranial nerves. Motor system was normal. Deep tendon reflexes were depressed but symmetrical bilaterally. Cerebellar testing showed no tremor and no dysmetria. There were no extrapyramidal signs. We found a horizontal and conjugate nystagmus with a frequency of 4 cycles per second. The nystagmus was increased by lateral gaze and by fixation attempts. It was composed of smooth eye movements of increasing velocity away from the target followed by refoveating saccades. The “null zone” was in the primary position, and there were no involuntary head movements (Video 2 in Supplementary Material available online at http://dx.doi.org/10.1155/2015/439409).

Routine laboratory tests and endocrine work-up were normal. EEG and MRI were normal.

### 2.3. Case 3

The patient was a 13-year-old male who presented with obesity, intellectual delay, bilateral visual impairment, and delayed pubertal signs. General physical examination showed mild mental retardation, awkward gait, emotional immaturity, retinitis pigmentosa, marked obesity, hypoplastic genitalia, and polydactyly (hexadactyly) in both feet. Neurological examination showed normal cranial nerves and motor system. Deep tendon reflexes were depressed but symmetrical. There were no extrapyramidal signs. Cerebellar testing was normal. A horizontal and conjugate nystagmus at a frequency of 4 cycles per second was present with the “null point” in the primary position. It consisted of a smooth movement of increasing velocity followed by a refoveating saccade (Video 3). There was no abnormal head posture. Routine blood tests, endocrine work-up, EEG, and MRI were all normal.

## 3. Discussion

The three siblings reported here presented with typical features of Laurence-Moon-Biedl (LMB) syndrome. A striking finding in all three was a binocular and conjugate horizontal nystagmus. Specifically, the nystagmus showed the characteristics of the “pseudocycloid” form of congenital nystagmus described by Dell'Osso and Daroff [3]. This type of nystagmus consists of a combination of smooth movements that are away from the intended target followed by refoveating and decelerating saccades. The smooth movement has an exponentially increasing velocity carrying the eyes away from the target, whereas the saccades refoveate or decelerate the eyes. According to Dell'Osso and Daroff [3] variations in the nystagmus morphology in null positions and other positions of gaze are attempts to increase foveation time. It is important to note that on gross examination this nystagmus may appear to be pendular in type, a common clinical misinterpretation in pseudocycloid nystagmus. However, a careful examination will reveal that the two phases of the nystagmus are not identical. Moreover, ENG studies in such cases will help distinguish between these two types of nystagmus [4]. We suspect that the previously reported instances of “pendular nystagmus” in LMB probably emanated from a misinterpretation of pseudocycloid nystagmus.

It should be noted that we did not perform electrophysiological studies (ENG) on our patients; hence, our interpretation of the nystagmus and the diagnosis of pseudocycloid nystagmus in our series were based on clinical grounds only.

The majority of patients with congenital nystagmus have abnormal posturing or tremors of the head to help improve their already impaired vision [5, 6]. However, such “compensatory mechanisms” did not occur in our case series. This could be explained by the fact that the “null point” in our patients was in the “primary gaze” position.

Oscillopsia (shimmering of objects) is occasionally reported by patients with congenital nystagmus associated with visual system defects (sensory-defect nystagmus) or without such defects (motor-defect nystagmus). The absence of oscillopsia (as observed in our patients) probably relates to the onset of nystagmus prior to visual-oculomotor maturity and the duration of nystagmus.

It is apparent from our study that the nystagmus in LMB is congenital and secondary to the associated retinitis pigmentosa (sensory-defect nystagmus). We conclude that this nystagmus is similar to idiopathic motor-defect nystagmus and the nystagmus seen in congenital optic nerve hypoplasia, ocular albinism, and bilateral opacities of the ocular media. Our data support the hypothesis of Yee et al. [7] that there is a common brain stem motor abnormality in sensory-defect and motor-defect nystagmus.

## Supplementary Material

A 13-year-old male with LMB syndrome and probable pseudocycloid nystagmus.

## Figures and Tables

**Figure 2:**